# Physiological, Ecological, and Biochemical Implications in Tomato Plants of Two Plant Biostimulants: Arbuscular Mycorrhizal Fungi and Seaweed Extract

**DOI:** 10.3389/fpls.2020.00999

**Published:** 2020-07-17

**Authors:** Mario Felipe González-González, Héctor Ocampo-Alvarez, Fernando Santacruz-Ruvalcaba, Carla Vanessa Sánchez-Hernández, Kena Casarrubias-Castillo, Amayaly Becerril-Espinosa, José Juvencio Castañeda-Nava, Rosalba Mireya Hernández-Herrera

**Affiliations:** ^1^Laboratorio de Investigación en Biotecnología, Departamento de Botánica y Zoología, Centro Universitario de Ciencias Biológicas y Agropecuarias, Universidad de Guadalajara, Zapopan, Mexico; ^2^Laboratorio de Ecología Molecular, Microbiología y Taxonomía, Departamento de Ecología, Centro Universitario de Ciencias Biológicas y Agropecuarias, Universidad de Guadalajara, Zapopan, Mexico; ^3^Laboratorio de Biotecnología Vegetal, Departamento de Producción Agrícola, Centro Universitario de Ciencias Biológicas y Agropecuarias, Universidad de Guadalajara, Zapopan, Mexico; ^4^Laboratorio de Marcadores Moleculares, Departamento de Producción Agrícola, Centro Universitario de Ciencias Biológicas y Agropecuarias, Universidad de Guadalajara, Zapopan, Mexico; ^5^CONACYT, Departamento de Ecología, Centro Universitario de Ciencias Biológicas y Agropecuarias, Universidad de Guadalajara, Zapopan, Mexico; ^6^Unidad de Biotecnología Vegetal, Centro de Investigación y Asistencia en Tecnología y Diseño del Estado de Jalisco (CIATEJ), Zapopan, Mexico

**Keywords:** plant biostimulant, *Rhizophagus intraradices*, *Padina gymnospora*, additive property, synergistic property, emergent properties

## Abstract

The worldwide use of plant biostimulants (PBs) represents an environmentally friendly tool to increase crop yield and productivity. PBs include different substances, compounds, and growth-promoting microorganism formulations, such as those derived from arbuscular mycorrhizal fungi (AMF) or seaweed extracts (SEs), which are used to regulate or enhance physiological processes in plants. This study analyzed the physiological, ecological, and biochemical implications of the addition of two PBs, AMF or SE (both alone and in combination), on tomato plants (*Solanum lycopersicum* L. cv. “Rio Fuego”). The physiological responses evaluated were related to plant growth and photosynthetic performance. The ecological benefits were assessed based on the success of AMF colonization, flowering, resistance capacity, nonphotochemical quenching (NPQ), and polyphenol content. Biochemical effects were evaluated *via* protein, lipid, carbohydrate, nitrogen, and phosphorous content. Each PB was found to benefit tomato plants in a different but complementary manner. AMF resulted in an energetically expensive (high ETR_MAX_ but low growth) but protective (high NPQ and polyphenol content) response. AMF + nutritive solution (NS) induced early floration but resulted in low protein, carbohydrate, and lipid content. Both AMF and AMF + NS favored foliar instead of root development. In contrast, SE and SE + NS favored protein content and root development and did not promote flowering. However, the combination of both PBs (AMF + SE) resulted in an additive effect, reflected in an increase in both foliar and root growth as well as protein and carbohydrate content. Moreover, a synergistic effect was also found, which was expressed in accelerated flowering and AMF colonization. We present evidence of benefits to plant performance (additive and synergistic) due to the interactive effects between microbial (AMF) and nonmicrobial (SEs) PBs and propose that the complementary modes of action of both PBs may be responsible for the observed positive effects due to the new and emerging properties of their components instead of exclusively being the result of known constituents. These results will be an important contribution to biostimulant research and to the development of a second generation of PBs in which combined and complementary mechanisms may be functionally designed.

## Introduction

The current challenges associated with horticultural production are growing due to the ever-increasing worldwide demand for efficient, environmentally friendly, and sustainable food production. Previously proposed variants of the term “Plant biostimulant” (PB) included biogenic stimulator (Filatov, 1951a; Filatov, 1951b), organic biostimulant (Russo and Berlyn, 1991), biostimulator (Goatley and Schmidt, 1991), biostimulant (Schmidt, 1992), and others as reviewed and cited in Yakhin et al. (2017). An *ad hoc* revision of PBs, which was published as “The Science of PBs - A bibliographic Analysis” (Du Jardin, 2012), was carried out by the European Commission. PBs were defined therein as highly heterogeneous materials that could be classified into eight categories: humic substances, complex organic materials, beneﬁcial chemical elements, inorganic salts, seaweed extracts (SEs), chitin and chitosan derivates, antitranspirants, and free amino acids and N-containing substances. However, this PB classification did not include any microbial biostimulants. Three years later, Colla and Rouphael (2015), in their special issue article titled “Biostimulants in horticulture,” proposed six nonmicrobial (i.e., chitosan, humic and fulvic acids, protein hydrolysates, phosphites, SEs, and silicon) and three microbial [i.e., arbuscular mycorrhizal fungi (AMF), plant growth-promoting rhizobacteria, and *Trichoderma* spp.] PBs. Thus, PBs are any applied substance or microorganism, including those of commercial products, which stimulate natural processes and improve nutrient uptake and use efficiency, abiotic stress tolerance, and crop quality (Du Jardin, 2015). However, PB definitions and classifications have recently been the focus of controversy. The new proposed deﬁnition of a PB is “a formulated product of biological origin that improves plant productivity as a consequence of the novel or emergent properties of the complex of constituents and not as a sole consequence of the presence of known essential plant nutrients, plant growth regulators, or plant protective compounds” (Yakhin et al., 2017). This particular conceptualization of PBs, in addition to allowing for a better understanding of their physiological and biochemical modes of action, has contributed to the development of PB science, industry, and legislation. Despite the notable progress in PB science in recent years, there are still many questions that remain open as well as many challenges and opportunities to identify patterns in complex data and elucidate the inherent activity and potential synergistic effects of the combination of microbial and nonmicrobial PBs for agricultural purposes (Rouphael and Colla, 2018).

The application of microbial and nonmicrobial PBs may efficiently improve yields without increasing the quantity of applied nutrients. Furthermore, the resultant effect of the combined application of microbial and nonmicrobial PBs may be antagonistic, additive, or synergistic. In antagonistic interactions, the combined effect of the PBs is lower than the sum of the PB effects when they are applied independently. In additive interactions, the combined effect of the PBs is equal to the sum of the PB effects when they are applied independently. Synergistic interactions occur when the combined effect of the PBs exceeds the sum of the PB effects when they are applied independently (Rouphael and Colla, 2018; Rouphael and Colla, 2020). However, knowledge of potential synergistic effects among PBs is scarce, and limited published data is available with respect to nutrient uptake efficiency or plant performance (Rouphael et al., 2017; Rouphael et al., 2018; Rouphael and Colla, 2018). Therefore, this study aimed to analyze the physiological, ecological, and biochemical implications of the addition of two PBs, AMF and SE, both independently and in combination, on the development of tomato plants (*Solanum lycopersicum* L. cv. “Rio Fuego”) to better elucidate the causal/functional mechanism of action.

AMF comprise an important microbial PB category composed of a soil biota functional group that has been found to positively affect crop production and support ecosystem sustainability (Rouphael et al., 2015a). The advantages plants derive from symbiosis go far beyond the nutritional benefits they obtain. Mycorrhizal plants have shown improved tolerance and resistance to a broad range of environmental stressors caused by both abiotic (e.g., drought or salinity) and biotic (e.g., pests and pathogens) factors due to the protection they gain from changes in various physiological parameters (Song et al., 2015; Begum et al., 2019 and literature cited therein). The mechanisms that mediate benefits from AMF are diverse and depend on the characteristics of the stress, and in most cases, these have been reported to be finely regulated by phytohormones (Pozo et al., 2015). Considering this complexity, further efforts are needed to unravel the mechanisms underlying the enhanced ability of host plants to overcome adverse conditions. This knowledge contributes to the promotion of the use of AMF as biostimulants and bioprotectors in agricultural practices as environmentally-friendly alternatives to traditional crop management strategies, which have generally depended on chemical fertilizer and pesticide application. AMF and plants live in symbiosis, and AMF hyphae grow into plant roots (Harrison, 2005; Gutjahr et al., 2009; Gutjahr and Paszkowski, 2013). Further, mycorrhizal ontogenesis has been linked to both host and symbiont growth and development (Chaudhary et al., 2019). Photosynthetic products are obtained from the host plant and utilized by fungi, who in turn supply the plant root system with soil nutrients, such as phosphorus, nitrogen, copper, and zinc (Ferrol et al., 2019). This form of symbiosis has been found to promote secondary metabolite synthesis, such as that of phenolic acids or flavonoids, which are essential for elevating abiotic stress tolerance in plants (Morandi, 1996; Strack and Fester, 2006; Schliemann et al., 2008; Tavarini et al., 2018). The economic and ecological value of AMF comes from approximately 80% of all land plants interacting with AMF, including agronomically important crops (Azcón-Aguilar and Barea, 1997). Recent studies using omics technologies have allowed researchers to further elucidate the important protective mechanisms resulting from AMF interactions that protect plants from abiotic stress, [i.e., Transcriptomics (Salvioli et al., 2012), Proteomics (Bernardo et al., 2017), and Metabolomics (Bernardo et al., 2019)].

The mechanism that has been proposed to explain the biostimulant activity of AMF with regard to plant performance is root biomass regulation that may enhance nutrient uptake and translocation, resulting in an increase in total carbohydrate, protein content, and phenolic levels while promoting growth, biomass production, stress tolerance, and disease resistance (Abbas, 2013; Colla et al., 2015a; Colla et al., 2015b; Rouphael et al., 2015a; Rouphael et al., 2017; Fiorentino et al., 2018; Rouphael and Colla, 2018; Lucini et al., 2019). In addition, AMF hyphal networks can enhance the quality of the soil by improving soil particle aggregation and reducing soil erosion by either wind or water. Further, AMF limit the amount of nutrients that are leached from the soil and thus promote nutrient retention while decreasing the risk of ground water contamination (Rouphael et al., 2015a; Chen et al., 2018; Tavarini et al., 2018).

Another important nonmicrobial PB category is based on SEs from brown, green, and red macroalgae, which have been proposed as sustainable amendments to improve crop yields without adverse environmental impacts (Khan et al., 2009). SEs are inexpensive and easy to prepare and use (Hernández-Herrera et al., 2014a). In addition, beneficial effects may be achieved with small SE doses (diluted to 1:1,000 or more; Crouch and van Staden, 1993). The active SE components have been identified as macroelements and microelements, such as nutrients, amino acids, vitamins, sugars (e.g., carbohydrates and oligo- and polysaccharides), growth hormones [e.g., cytokinins, auxins, gibberellins, and abscisic acid (ABA)-like growth substances], or low-weight molecular components (e.g., polyamines and brassinosteroids), all of which have been found to affect cellular metabolism and enhance crop growth and yield (Khan et al., 2009; Battacharyya et al., 2015; Shukla et al., 2019). Furthermore, SE components that are present in moderate or large quantities, such as polyphenols (e.g., phloroglucinol and eckol) or polysaccharides (e.g., alginate, fucoidan, laminarian, carrageenan, and their derived oligosaccharides), have also been found to promote plant growth (Hong et al., 2007; Khan et al., 2009; Craigie, 2011; González et al., 2013; Battacharyya et al., 2015; Rengasamy et al., 2015a; Rengasamy et al., 2015b; Hernández-Herrera et al., 2016; Mzibra et al., 2020).

SEs also provide an alternative means to manage pests and prevent plant disease (Baloch et al., 2013), increase plant tolerance to abiotic stress (EL Boukhari et al., 2020), and improve the number of fruits or other quality traits (Hamed et al., 2018). These are a result of the combined actions of various complex pools of bioactive molecules found within SEs (Khan et al., 2009; Ertani et al., 2018). According to Shukla et al. (2019), a specific mode of action for SEs is the role they play in plant growth by regulating genes involved in nutrient acquisition and thus enhancing nutrient uptake.

We hypothesized that the independent application of AMF and SE would be favorable but would differ with regard to plant development. However, we assumed that when microbial and nonmicrobial PBs were used in combination, their combined effect on the plants would be far superior (synergistic) to that of either PB when applied independently due to the new and emerging properties of the constituent complex.

## Materials and Methods

### Experimental Design and Plant Material

The greenhouse experiment was performed in a complete randomized block design with a total of six treatments that contained 12 experimental units (replicas) each (n = 72 plants). The experiment was carried out from February to May in 2019. The treatments consisted of (1) plants grown without any PBs and only irrigated with Rorison nutritive solution (NS); (2) plants treated with a microbial-based biostimulant containing *Rhizophagus intraradices*, a type of AMF; (3) plants treated with a microbial-based biostimulant containing AMF and irrigated with the NS (AMF + NS); (4) plants treated with a nonmicrobial-based biostimulant from a *Padina gymnospora* extract (SE); (5) plants treated with a nonmicrobial-based biostimulant from SE and irrigated with the NS (SE + NS); and (6) plants grown with both PBs and irrigated with the NS (AMF + SE + NS). A control group of plants irrigated with only water was also included, although the plants did not survive until the end of the experiment, and thus the data are not shown.

*Solanum lycopersicum* L. cv. “Rio Fuego” seeds (Kristen Seed, San Diego, USA) were surface-sterilized in 3% sodium hypochlorite for 10 min, triple-rinsed in sterile distilled water, and planted individually in 1-L pots with a sterile soil mixture composed of vermiculite:sand (1:2, v/v) that had been autoclaved thrice. The plants were cultivated under natural light conditions. Daily temperature in the greenhouse was always maintained below 27°C ± 2°C, and the night temperature was always higher than 15°C ± 2°C. The average day/night relative humidity was ~ 85%.

### PB Application

The AMF microbial-based biostimulant containing *Rhizophagus intraradices* (N.C. Schenck and G.S. Smith) C. Walker and A. Schüßler, previously referred to as *Glomus intraradices*, was produced by Experimental Field Bajio at the National Research Institute of Forestry, Agriculture, and Livestock (INIFAP). The *R. intraradices* AMF was comprised of a mixture of mycelia, root segments, spores, and soil-sand and is commercialized as Mycorrhiza-INIFAP^®^ (INIFAP^®^, Celaya, Guanajuato, Mexico).

The SE was obtained from the Biotechnology Research Laboratory of the Universidad de Guadalajara (Guadalajara, Mexico). The methodology for its preparation followed that of Hernández-Herrera et al. (2014a; 2014b). Briefly, 8 g of dry powder from the brown seaweed *Padina gymnospora* (Kützing) Sonder was added to 1 L of distilled water, constantly stirred for 15 min, and autoclaved at 121°C for 1 h at 1.21 kg cm^-2^. The hot extract was filtered through Whatman No. 40 filter paper from Sigma-Aldrich (Merck KGaA, Darmstadt, Germany) and stored at −4°C until further use. The chemical composition of the *P. gymnospora* SE at a 0.8% concentration was analyzed, and the results are shown in **Supplementary Table S1**.

For the experiment, the tomato seeds were sown in sand and divided into six groups. Two groups of seeds were first treated by covering them with a microbial-based biostimulant solution of AMF at a concentration of 3 g L^-1^ (~ 100 spores g^-1^) and then watered (one group with NS and the other with only water). Similarly, two other groups of tomato seeds were treated with the nonmicrobial biostimulant, SE. For the nonmicrobial PBs treatment, the SE was added directly to the substrate (50 mL of *P. gymnospora* at a concentration of 0.8%) on planting day (one SE group was watered with NS and the other with only water). Furthermore, a group of tomato seeds was treated with a combination of both biostimulants (AMF and SE) and then watered with NS. In all treatments, the initial application of the biostimulant took place on planting day with an additional application on day 15 when the tomato seedlings had already emerged. After which, watering took place every two weeks for a total of five applications of the PBs during the experiment. The control treatment was comprised of seeds that were planted in sand without PB and only watered with the NS. All of the plants of the treatments that were watered with NS were irrigated weekly with the NS known as Rorison nutrient solution (Hewitt, 1966) with modifications. The phosphate concentration of the solution was diluted to 0.05 mM to decrease the KH_2_PO_4_ content to favor AMF colonization. The composition of the Rorison NS used in this work is shown in **Supplementary Table S2**. Furthermore, all plants in all treatments were watered with additional distilled water according to their needs.

### Physiological Responses of Tomato Plants

After 96 d, the effects of AMF and SE on plants were analyzed by measuring physiological descriptors. Initially, photosynthetic performance was measured in six randomly selected plants per treatment *via* nonintrusive pulse amplitude modulated chlorophyll fluorometry using a Junior PAM fluorometer (Heinz Walz GmbH, Effeltrich, Germany). The leaves were dark-acclimated with leaf clips for 20 min before the start of a rapid light curve (RLC) routine. Actinic illumination in the RLC trial was increased in 12-step increments from 5-1500 *μ*mol photon m^−2^ s^−1^ with a total of 30 s for each light level. The maximum electron transport rate (ETR_MAX_) was calculated from the RLC according to the methodology of Murchie and Lawson (2013) and used as a proxy for plant photosynthetic performance. In the same fluorescence trial, the maximum photochemical quantum yield efficiency of PSII in a dark-adapted state (*F*_*V*_/*F*_*M*_) and nonphotochemical quenching (NPQ) were also obtained to evaluate the photoinhibition state and photoprotection capacity of the plants due to PB addition.

Once the chlorophyll fluorescence parameters were measured in the living plants, the plants were harvested to evaluate their growth characteristics. The plants were carefully removed from their plastic pots and submerged immediately in bowls filled with water at ~20°C for 20 min. Then, the 12 plants per treatment were photographed and growth characteristics were measured using ImageJ v. 1.52a software (https://imagej.nih.gov/ij/download.html). Data were used to obtain the ratio of the projected shoot, root, and total length; leaf and root area; and the number of leaves and flowers. After growth was measured, the root and foliar system were also carefully washed to eliminate sand particles and subsequently dried with blotting paper for further analysis.

### Biochemical Characteristics of Tomato Plants

The effects of AMF and SE on plants were evaluated by measuring the biochemical composition of the 12 replicas per treatment (n = 12 tomato plants). The samples were oven dried at 65°C for 72 h. A 100-g sample of dry material was used for quantifying protein, lipid, total carbohydrate, nitrogen, phosphorous, and total polyphenol content. The methods followed those of the Association of Official Analytical Chemists (A.O.A.C., 1990) for lipids (954.04), nitrogen (955.04), and proteins using a factor of 6.25 (954.04). Phosphorous determination was performed according to Mengel and Kirkby (1987), and total carbohydrate content was evaluated with the DuBois method (DuBois et al., 1956), which included a standard glucose calibration curve (Merck KGaA). Finally, the Folin-Ciocalteu colorimetric method, based on the procedure of Singleton and Rossi (1965), was used to estimate total polyphenol content using gallic acid as the standard. All chemical measurements were performed in triplicate (each replicate consisted of a mix of four plants). A separate sub-sample of the roots per plot was frozen immediately with liquid nitrogen and kept at −80°C for subsequent gene expression analyses.

### Ecological Benefits and AMF Molecular Colonization in Tomato Plants

The success of the mutual association between AMF and tomato plants was determined at the end of the experiment based on root measurements. Root systems were washed in cold water and their fresh weights were recorded. Small root samples were stored in liquid nitrogen for RNA extraction, and a fresh root fraction was ﬁxed in formalin/acetic acid/ethanol (FAE, 13:5:200 [v/v/v]) for 24 h to determine the degree of AMF colonization. The roots were cut into 1-cm pieces and then placed in 10% KOH at 99°C for 1.5 h. The samples were then stained with 0.05% (w/v) trypan blue in lactophenol following the methods described by Phillips and Hayman (1970). The AMF were examined under a Primo Star compound light microscope (Carl Zeiss, Gottingen, Germany). Fungal colonies were estimated as described by Trouvelot et al. (1986) using the MYCOCALC program^1^ by means of the AMF colonization in the root system (M%) and arbuscule abundance (A%) quantiﬁed from six randomly selected roots per treatment (each one with 30 replicates). A total of 180 fragments from each treatment group were evaluated.

In addition, another sub-sample of the same six roots per treatment was immediately frozen with liquid nitrogen and kept at −80°C for subsequent gene expression analyses. The expression of symbiosis marker genes (i.e., *RiEF* and *LePT4)* was analyzed to confirm mycorrhizal colonization. Total RNA was extracted from the roots obtained from a pool of six plants using Trizol reagent (Invitrogen, Carlsbad, USA) according to the protocol of the manufacturer. cDNA was obtained from 2 μg of RNA using M-MLV reverse transcriptase (Promega, Madison, USA) and oligo-dT (12–18) primers. To determine gene expression, real-time quantitative PCR (RT-qPCR) amplifications were performed in 96-well plates using SYBR Green (Thermo Fisher Scientific, Germantown, USA) detection chemistry in a StepOnePlus™ RT-qPCR System (Applied Biosystems, Foster City, USA). Reactions were prepared in a total volume of 15 μl, with 2 μl of cDNA template (1:10), 7.5-μl SYBR^®^ Select Master Mix (Applied Biosystems), and forward and reverse primers (300 nM). The gene-specific primers were designed from GenBank sequences (**Supplementary Table S3**). The cycling conditions were set as follows: initial denaturation step at 95°C for 5 min, followed by 40 cycles of denaturation at 95°C for 10 s, and annealing at 60°C for 30 s. A melting curve analysis was used to evaluate reaction specificity. The baseline and cycle threshold (Ct) were automatically determined using the RT-qPCR system software. Relative expression was calculated using a comparative cycle threshold method (Livak and Schmittgen, 2001). Transcript abundance was normalized using the housekeeping gene of the SAND family proteins as an endogenous reference (Expósito-Rodríguez et al., 2007).

### Statistical Analysis

Twelve plants per treatment were used for each analysis (mean ± SD). An analysis of variance (ANOVA) was performed to evaluate significant differences among treatments for all physiological, ecological, and biochemical descriptors with Statgraphics Centurion XV (Statpoint Technologies, Inc., The Plains, USA). Significance (*P* ≤ 0.05) was identified with the general linear model (GLM) procedure and the least significant difference (LSD) mean comparison test. A joint principal coordinates analysis (PCoA) was performed on the physiological variables to evaluate their interdependence, including photosynthetic performance (ETR_MAX_) and growth parameters (shoot, root, and total length and leaf and root area). A second PCoA analysis was performed for biochemical and ecological benefits (i.e., the success of the mutual association *via* AMF colonization), flowering, resistance capacity (NPQ), and polyphenol content. In addition, a Cluster-Simprof analysis was performed on the Euclidian distance matrixes constructed from square root-transformed descriptor data to identify similar patterns among the PB treatments. The cluster groups, PCoA figures, and correlation values were generated using Primer 7 + Permanova (PRIMER-E Ltd., Plymouth, UK) according to the methodology of Anderson et al. (2008).

## Results

### Physiological Characteristics of Tomato Plants in Response to PB Addition

**Figure 1:**
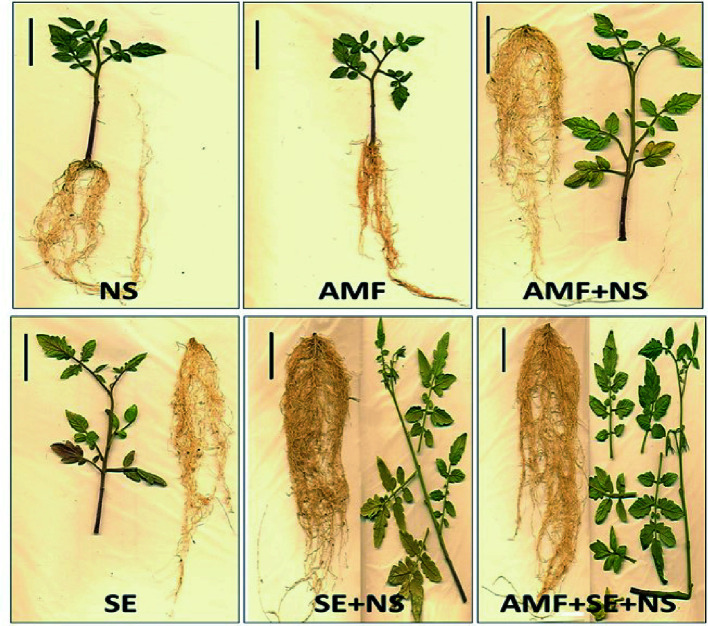
Tomato plant growth after the 96-day experiment. Treatments included plants irrigated with nutritive solution (NS), treated with a microbial-based biostimulant containing *Rhizophagus intraradices* [arbuscular mycorrhizal fungi (AMF)], treated with an AMF and irrigated with the nutritive solution (AMF + NS), treated with not microbial-based biostimulant from *Padina gymnospora* extract [seaweed extract (SE)], treated with SE and irrigated with nutritive solution (SE + NS), and a combination of both plant biostimulants plus irrigation with nutritive solution (AMF + SE + NS). Bar, 5 cm.

**Figure 2:**
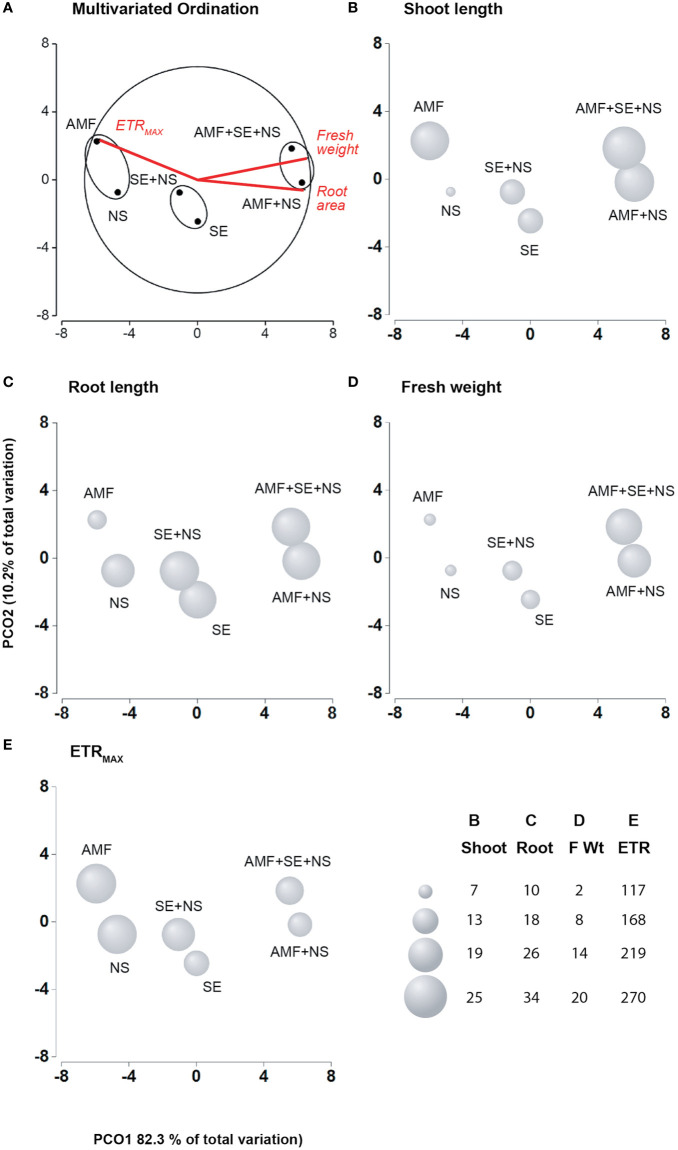
Principal coordinates analysis (PCoA) of the physiological characteristics of the tomato plants irrigated with nutritive solution (NS), treated with a microbial-based biostimulant containing *Rhizophagus intraradices* [arbuscular mycorrhizal fungi (AMF)], treated with an AMF and irrigated with the nutritive solution (AMF + NS), treated with not microbial-based biostimulant from *Padina gymnospora* extract [seaweed extract (SE)], treated with SE and irrigated with nutritive solution (SE + NS), and a combination of both plant biostimulants and irrigated with nutritive solution (AMF + SE + NS). **(A)** Multivariate Ordination. **(B)** Shoot length**. (C)** Root length. **(D)** Fresh weight. **(E)** Maximum electron transport rate (ETR_MAX_; n = 6 plants).

The physiological parameters of photosynthesis and growth performance were significantly (*P* ≤ 0.05) affected by AMF and SE PBs as well as the combination of AMF and SE (**Tables 1** and **2**). **Figure 1** shows an example of a growth image pertaining to a representative plant of each PB treatment. A biplot analysis was used to confirm the relationship that was expressed in the ANOVA among the PBs and the physiological parameters of the tomato plants. The PCoA graphs in **Figures 2A–E** show a comparison among the physiological parameters of the PB-treated plants. Two factors explained 92.5% of the total variance. Factor 1 (PCO1) explained 82.3% of the variance and was negatively correlated with root area and fresh weight, while also being positively correlated with ETR_MAX_ (**Supplementary Table S4**). Factor 2 (PCO2) explained 10.2% of the variance and was positively correlated with shoot length. By plotting data according to PCO1 and PCO2, three clusters were identified that showed a clear separation among tomato plants from the different PB treatments.

**Table 1:** Growth of tomato plants treated with plant biostimulants.

Treatments	Length (cm)			Area (cm^2^)		Weight (g)			Total Flowers
	Shoot	Root	Total	Leaf	Root	Fresh		Dry	
NS	16.9 ± 1.2^b^	24.4 ± 8.2^b^	39.1 ± 9.1^a^	11.3 ± 2.3^a^	43.4 ± 13.8^b^	1.65 ± 0.51^a^		0.71 ± 0.32^b^	0
AMF	21.1 ± 4.1^c^	18.0 ± 1.3^a^	39.1 ± 4.6^a^	23.8 ± 8.1^b^	11.5 ± 4.9^a^	1.67 ± 0.49^a^		0.18 ± 0.08^a^	0
AMF + NS	21.9 ± 1.9^c^	31.5 ± 5.1^c^	53.6 ± 8.6^d^	104.3 ± 4.3^d^	143.1 ± 28.4^d^	12.65 ± 1.86^c^		1.30 ± 0.80^cd^	5
SE	12.5 ± 3.6^a^	30.8 ± 5.3^c^	45.4 ± 6.1^c^	31.8 ± 9.8^c^	50.9 ± 8.4^b^	4.13 ± 0.84^ab^		1.23 ± 0.23^c^	0
SE + NS	12.4 ± 2.3^ab^	33.8 ± 5.2^c^	44.7 ± 5.4^bc^	34.8 ± 6.2^c^	58.6 ± 11.2^b^	4.32 ± 0.93^b^		1.31 ± 0.26^cd^	0
AMF + SE + NS	24.8 ± 2.0^d^	32.5 ± 2.4^c^	58.4 ± 2.9^e^	120.5 ± 20.3^e^	117.8 ± 5.03^c^	14.46 ± 1.65^d^		1.56 ± 0.29^d^	13

Tomato plants irrigated with nutritive solution (NS), treated with a microbial-based biostimulant containing Rhizophagus intraradices (AMF), treated with a AMF and irrigated with the nutritive solution (AMF + NS), treated with not microbial-based biostimulant from Padina gymnospora extract (SE), treated with SE and irrigated with nutritive solution (SE + NS), and a combination of both plant biostimulants and irrigated with nutritive solution (AMF + SE + NS). Values are mean ± standard deviation (n = 6. Different letter (a–d) within the columns indicate significant differences according to the least significant differences (LSD) mean comparison test (p = 0.05).

**Table 2:** Maximum electron transporter rate (ETR_MAX_), photochemical quantum yield of PSII (*F*_*V*_ */ F*_*M*_), and nonphotochemical quenching (NPQ) measured in tomato plants treated with plant biostimulants.

Treatments	ETR_MAX_	*F*_*V*_*/F*_*M*_	NPQ
NS	262.00 ± 15.87^d^	0.802 ± 0.00^b^	0.84 ± 0.10^a^
AMF	268.84 ± 32.9^d^	0.801 ± 0.01^b^	1.30 ± 0.10^b^
AMF + NS	104.98 ± 9.07^a^	0.757 ± 0.00^a^	0.99 ± 0.11^a^
SE	112.64 ± 14.93^a^	0.778 ± 0.02^ab^	0.81 ± 0.15^a^
SE + NS	197.63 ± 31.14^c^	0.786 ± 0.01^a^	0.99 ± 0.14^b^
AMF + SE + NS	151.92 ± 13.14^b^	0.783 ± 0.01^a^	0.96 ± 0.25^b^

Tomato plants irrigated with nutritive solution (NS), treated with a microbial-based biostimulant containing Rhizophagus intraradices (AMF), treated with a AMF and irrigated with the nutritive solution (AMF + NS), treated with not microbial-based biostimulant from Padina gymnospora extract (SE), treated with SE and irrigated with nutritive solution (SE + NS), and a combination of both plant biostimulants and irrigated with nutritive solution (AM F+ SE + NS). Values are mean ± standard deviation (n = 6). Different letters (a–d) within the columns indicate significant differences according to the least significant difference (LSD) mean comparison test (p = 0.05).

**Figure 3:**
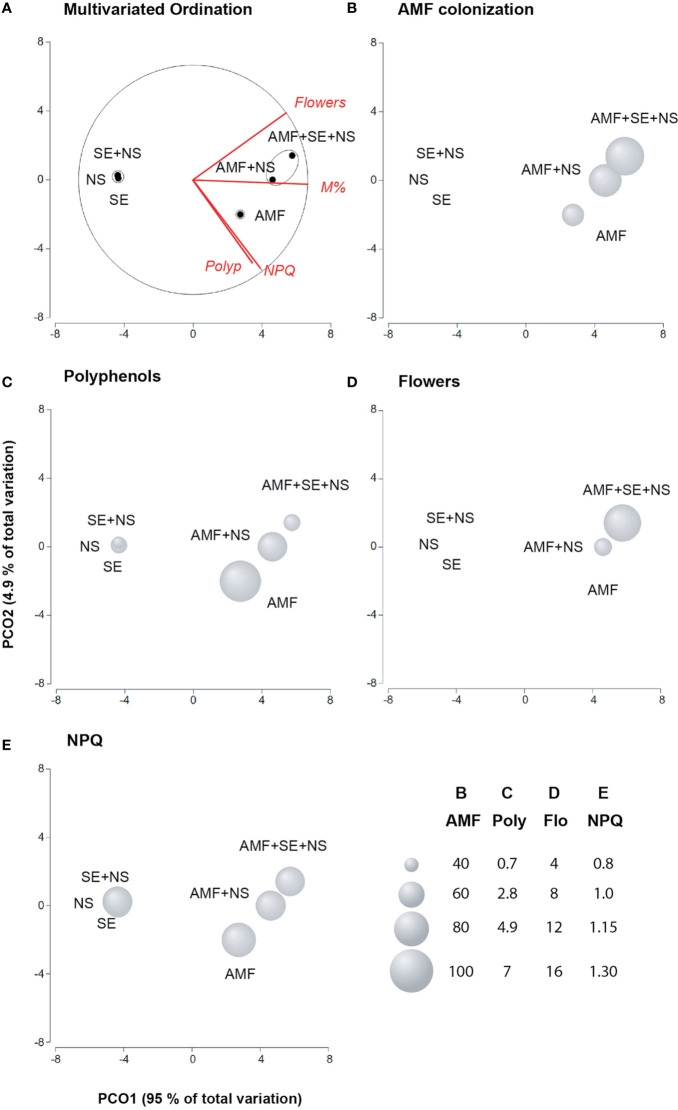
Principal coordinates analysis (PCoA) of the ecological benefits of tomato plants irrigated with nutritive solution (NS), treated with a microbial-based biostimulant containing *Rhizophagus intraradices* [arbuscular mycorrhizal fungi (AMF)], treated with a AMF and irrigated with the nutritive solution (AMF + NS), treated with not microbial-based biostimulant from *Padina gymnospora* extract [seaweed extract (SE)], treated with SE and irrigated with nutritive solution (SE + NS), and a combination of both plant biostimulants and irrigated with nutritive solution (AMF + SE + NS). **(A)** Multivariate ordination; **(B)** AMF colonization of the root system; **(C)** Polyphenols; **(D)** Flowers, **(E)** Nonphotochemical quenching (NPQ; n = 6 plants).

The first group was composed of plants treated with AMF and plants irrigated with NS. The AMF-treated plants presented the lowest root length growth and fresh weight, although their shoot length was significantly larger than that of the NS plants (*p* ≤ 0.05), highlighting the biostimulant activity of AMF. Moreover, AMF conferred an extremely high resistance to environmental stress in their host plants, as determined from the high polyphenol content as well as the high NPQ and *F_V_/F_M_* values (**Tables 2** and **3**, **Figure 3E**) that reflected an augmentation of antioxidant and photoprotective mechanisms. In contrast to the AMF plants, the NS plants that grew without PB addition but that were irrigated with the NS presented low leaf growth yet large and heavy roots (**Table 1**) as well as significantly lower NPQ values (**Table 2**). This result indicates that the NS plants presented lower antioxidant and photoprotective capacity than that of the AMF plants.

**Table 3:** Biochemical content of tomato plants treated with plan biostimulants.

Treatments	Protein (%)	Lipids (%)	Carbohydrates (mg·g^−1^)	Polyphenols (mg·g^−1^)	Nitrogen (%)	Phosphorous (mg·Kg^−1^ P)
NS	10.81 ± 0.41^b^	4.51 ± 0.27^c^	4.672 ± 0.35^e^	0.050 ± 0.01^a^	1.2 ± 0.01^a^	0.119 ± 0.05^b^
AMF	10.58 ± 0.19^b^	2.44 ± 0.13^b^	2.134 ± 0.39^b^	0.687 ± 0.01^d^	2.3 ± 0.01^b^	0.014 ± 0.05^a^
AMF + NS	9.29 ± 0.16^a^	2.73 ± 0.23^b^	3.750 ± 0.16^d^	0.352 ± 0.01^c^	2.1 ± 0.01^b^	0.223 ± 0.05^c^
SE	12.20 ± 0.21^c^	2.46 ± 0.11^b^	0. 952 ± 0.01^a^	0.108 ± 0.02^b^	1.9 ± 0.01^a^	0.080 ± 0.05^b^
SE + NS	10.44 ± 0.24^b^	5.49 ± 0.12^d^	2.944 ± 0.14^c^	0.057 ± 0.01^a^	2.1 ± 0.01^b^	0.100 ± 0.05^b^
AMF + SE + NS	13.22 ± 0.19^d^	1.42 ± 0.36^a^	5.164 ± 0.22^f^	0.114 ± 0.00^b^	1.8 ± 0.01^a^	0.201 ± 0.05^c^

Tomato plants irrigated with nutritive solution (NS), treated with a microbial-based biostimulant containing Rhizophagus intraradices (AMF), treated with a AMF and irrigated with the nutritive solution (AMF + NS), treated with not microbial-based biostimulant from Padina gymnospora extract (SE), treated with SE and irrigated with nutritive solution (SE + NS), and a combination of both plant biostimulants and irrigated with nutritive solution (AMF + SE + NS). Values are mean ± standard deviation (n = 3, each replicate is a mix of four plants). Different letters (a–d) within the columns indicate significant differences according to the least significant difference (LSD) mean comparison test (p = 0.05).

As no differences in the degree of photoinhibition were present between the AMF and NS plants, which was evident in their similar *F_V_/F_M_* values, it appears that the high ETR_MAX_ energy in NS plants may have been spent repairing photosynthetic machinery and developing larger root systems to obtain missing nutrients. The second group was composed of SE-treated plants (SE) and SE-treated plants irrigated with the NS (SE + NS). This group exhibited greater shoot and root length, root surface area, and fresh weight than those of the AMF and NS plants. These results highlight the PB power of SE. Interestingly, a significant beneficial effect was observed in the third group that included AMF-treated plants irrigated with the NS (AMF + NS) as well as AMF- and SE-treated plants irrigated with the NS (AMF + SE + NS). This third plant group displayed the highest growth values and a down-regulation of the electron transport rate at the PSII level (ETR_MAX_), which suggests an optimization of energetic resources (**Figures 2A–E**).

### Biochemical and Ecological Effects in Response to PB Addition

By relating plant parameters with AMF and SE PBs through a PCoA, two factors were found to explain 99.9% of the total variance (**Figures 3A–E**). Factor 1 (PCO1) explained 95% of the variance and was negatively correlated with AMF colonization in the root system (M%) and the number of flowers. Factor 2 (PCO2) explained only 4.9% of the variance and was positively correlated with polyphenol content and NPQ values (**Supplementary Table S5**).

Plotting data according to PC1 and PC2 resulted in three clusters that were clearly separated by the PB properties of the different treatments. Group 1 was composed of NS plants, SE-treated plants, and SE + NS plants. These plants showed the lowest polyphenol content, low NPQ induction, and no flower development. Logically, no mycorrhiza colonization was observed. Cluster 2 was composed of AMF-treated plants. These plants showed the lowest AMF colonization in the root system but presented the most effective NPQ induction and the highest polyphenol content. Increasing NPQ may play a key role in dissipating excess energy to prevent the photosynthetic machinery from being destroyed by excess light energy. Polyphenols may also help to destroy ROS molecules that affect the PS II photosynthetic core protein D1. Therefore, AMF treatment alone conferred ecological advantages to the tomato plants under low nutrient conditions (**Figures 3A–E**). Finally, the third cluster included AMF + NS plants and AMF + SE + NS plants. This final group of plants displayed high AMF root colonization as well as the greatest biomass yield and number of flowers (**Figures 3A–E**).

With respect to the biochemical composition of the tomato plants treated with the combination of both PBs and irrigated with the NS (AMF + SE + NS), these plants showed the greatest yield with regard to both fresh and dry biomass as well as high protein, carbohydrate, and phosphorous content in leaf tissues (**Tables 1** and **3**).

### Root Mycorrhizal Colonization

**Figure 4:**
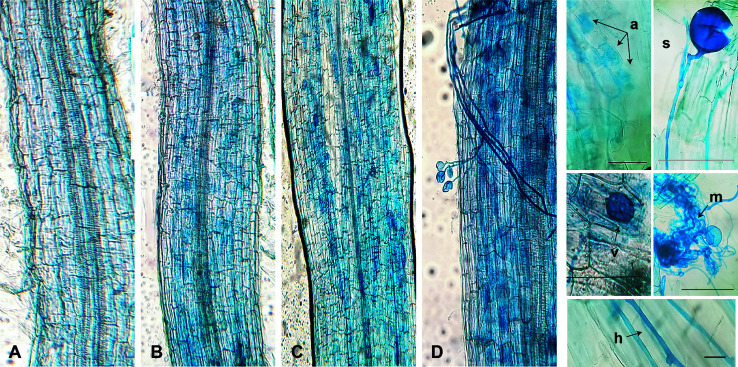
Optical micrographs of the tomato roots and morphological structures of *Rizophagus intraradices* during mycorrhizal symbiosis **(A)** Non-arbuscular mycorrhizal fungi (AMF) in root, **(B)** AMF in root, **(C)** AMF in root and irrigated with nutritive solutions (AMF + SN), and **(D)** AMF in root with seaweed extract and irrigated with nutritive solution (AMF + SE + NS). Spore (s), hyphae (h), mycelium (m), arbuscule (a), vesicle (v). Scale bars represent 200 µm 40×. (n = 6 plants).

Plants were collected 96 d after AMF treatment. Fungal structures were stained within the roots and showed well-established mycorrhizal symbiosis in the AMF-treated and AMF + SE + NS plants, with ample fungal colonization and well-formed arbuscules at the root cortex. The absence of fungal structures was conﬁrmed in the roots of the plants from the nonmycorrhizal treatments (**Figure 4**). The AMF colonization in the root system (91.5%), arbuscule abundance (84%), and vesicle number (125) were more abundant in the roots of plants treated with the combination of both PBs and irrigated with the NS (AMF + SE + NS). The AMF colonization levels agreed with the expression level of the *RiEF1-α* gene marker of *Rhizophagus intraradices*. Gene expression was elevated in AMF + NS plants and slightly more so in AMF + SE + NS plants (**Table 4**).

**Table 4:** *Rizophagus intraradices* colonization measured on the tomato root system. AMF colonization of the root system (M%) and arbuscule abundance (A%) and relative expression values of symbiosis marker genes.

Treatment	Spores	Hyphae	Mycelium	Vesicles	M%	A%	*Ri EF-1α*	*LePT4*
AMF	2	128	27	70	52.2	44.6	1.09 ± 0.6	1.00 ± 0.0
AMF + NS	6	144	40	112	74.0	67.9	15.09 ± 2.8	0.99 ± 0.8
AMF + SE + NS	0	163	120	125	91.5	84.0	21.14 ± 2.5	1.85 ± 0.76

Plants treated with arbuscular mycorrhiza fungi (AMF), treated with AMF and irrigated with nutritive solution (NS) and combination of both microbial (AMF) and nonmicrobial seaweed extract (SE) and watered with nutritive solution (AMF + SE + NS). Data shown n = 6. Expression values of AMF symbiosis marker genes (mean ± standard deviation).

Functional symbiosis was evaluated by analyzing *LePT4* expression. In arbuscule-containing cells in which a notable proportion of nutrient exchange occurs, the *LePT4* phosphate transporter is induced, and thus *LePT4* expression may be used to evaluate functional symbiosis. No difference was observed in AMF-treated or AMF + NS plants, and *LePT4* expression was similar among treatments. However, when plants were treated with a combination of AMF and SE and irrigated with NS (AMF + SE + NS), a strong induction of *LePT4* expression was detected (1.8-fold greater than that of AMF + NS plants), indicating that adding SE promoted AMF development, mycorrhizal establishment, and symbiosis (**Table 4**).

## Discussion

In recent years, a lot of agricultural research has focused on the discovery of environmentally friendly management strategies that ensure food safety. The application of PBs has been one of the best strategies to fulfill these terms, and consequently the use of biostimulants has evolved over time to produce increasingly better results with regard to plant performance. The first generation of biostimulants (1.0) was composed of products created from bioactive substances and/or microorganisms found in organic materials. These products improved nutrient uptake and use efficiency by stimulating physiological and molecular processes, which enhanced tolerance to abiotic stress and increased product yield and quality (Du Jardin, 2015). Then, efforts were directed to the discovery, development, characterization, and production of new PBs that were very different from those of generation 1.0. The new obtained PBs allowed researchers to identify the components of a successful PB formulation by elucidating complex biomolecular modes of action and identifying new opportunities for the creation of novel or improved PBs (Povero et al., 2016). With this approach, natural substance functions and the manner in which they modulate plant physiology may be predicted, resulting in the development of PBs that perform well under diverse conditions, including under stress (De Pascale et al., 2017; Parađiković et al., 2019). This approach relies on a powerful combination of chemistry, biology, and omics to identify patterns in complex data and elucidate the inherent activity and synergy of potential microbial and nonmicrobial PBs for commercial agricultural applications (Rouphael and Colla, 2018). The synergistic effects among microbial and nonmicrobial biostimulants, including those of different origins, have enabled researchers to design and formulate efficient PB products (2.0) with specific yield characteristics, particularly with regard to nutritional and functional qualities (Rouphael and Colla, 2018).

**Figure 5:**
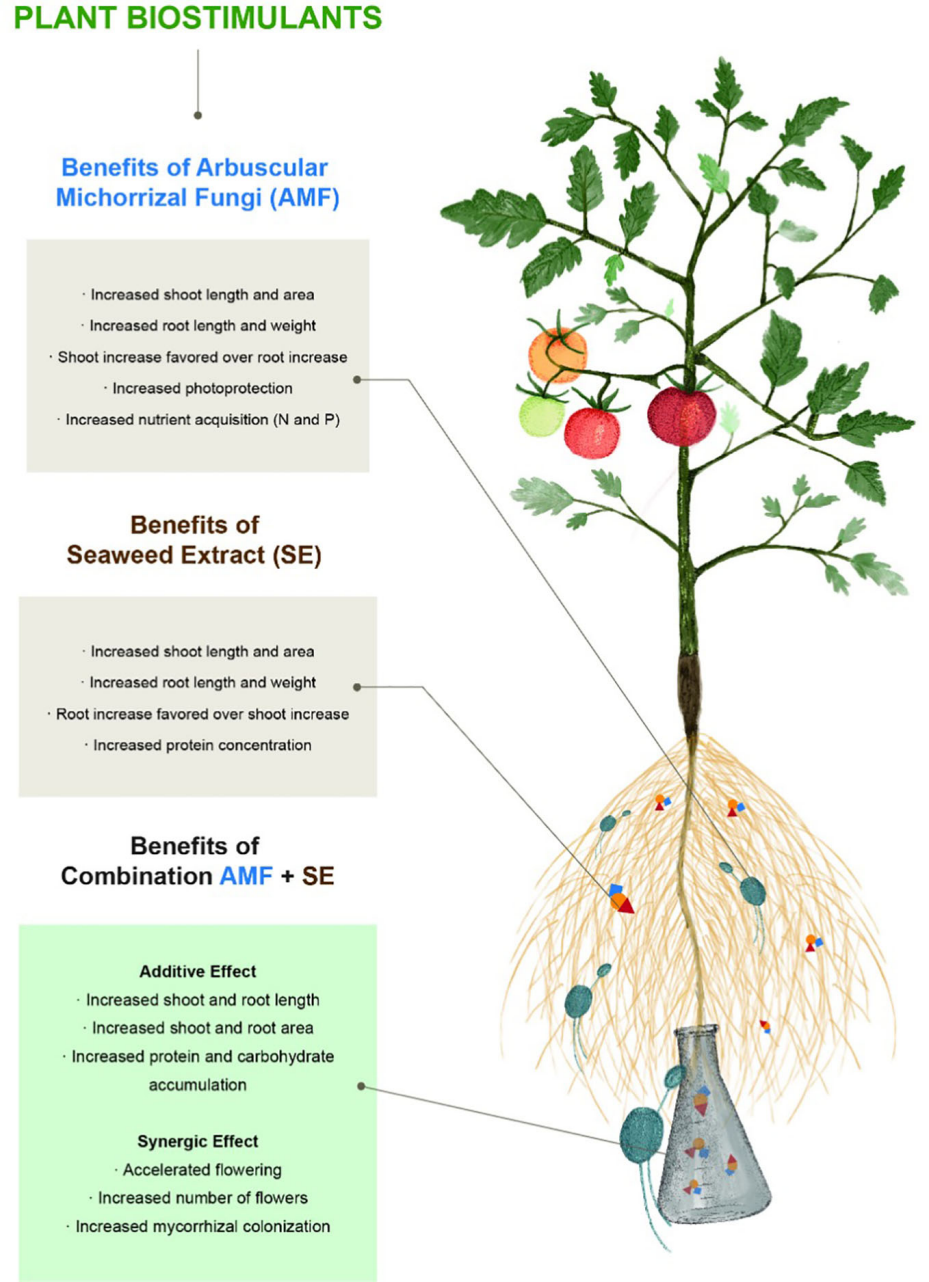
Plant biostimulants contributions of arbuscular mycorrhizal fungi (AMF) and seaweed extract (SE), both alone and in combination on tomato plants (*Solanum lycopersicum* L. cv. “Rio Fuego”).

In this work, we found that each PB positively affected the physiological, ecological, and biochemical composition of the tomato plants in different but complementary ways (**Figure 5**). Initially, we analyzed the physiological responses in the plants in each PB treatment. For example, AMF conferred specific benefits related to plant physiology, including enhanced plant growth (preferential growth in the aerial portion instead of the plant root) and development (accelerated floration). Numerous studies have reported root system changes in response to AMF that have promoted root branching and increased the volume of the root system (reviewed in Gutjahr and Paszkowski, 2013; Pozo et al., 2015). Root-associated AMF trigger root biomass increases that have been attributed to increased auxin levels. A mechanism has been proposed to explain this in which mycorrhizal symbiosis modifies the development of the plant structure below the ground, including affecting the timing, extent (e.g., number and volume of lateral roots and root hairs), and degree (e.g., secondary and tertiary roots) of root branching during primary root growth. This can be simply summarized by auxin inhibiting root elongation while lateral root development is strongly promoted (Sukumar et al., 2013). All of these effects are believed to change the hormonal state of the plant and consequently increase nutrient assimilation (Yakhin et al., 2017). Rapid plant growth depends on the ability of roots to provide sufficient water and nutrients to meet the requirements of acquisitive leaves with high photosynthetic rates and evaporative demands (Reich, 2014). However, our results show that the complete mechanisms are much more complex since root and foliar biomass varied according to nutrient conditions, in what seemed to be mycorrhiza-plant coordination.

Other important beneﬁcial eﬀects of microbial biostimulants have been frequently associated with improved photosynthetic apparatus functioning and pigment biosynthesis (Colla et al., 2015b and references cited therein; Rouphael et al., 2015b). However, as Demmig-Adams et al. (2017) recently discussed, photosynthesis and photosynthetic parameters change in many ways according to the response of the organism as a whole to environmental or even ecological conditions. Therefore, we consider that the photosynthetic responses of our treatments should not only be taken as either high or low with regard to the PBs added but as the response of the whole organism and the PB to the environmental conditions of the experiment, such as the presence of NS. In our work, the plants grown under low-nutrient conditions and treated with AMF showed high ETR_MAX_ values that were not reflected in plant growth. This implies that arbuscular mycorrhization promoted PSII photochemistry in low nutrient soil. Nevertheless, the fixed carbon in the plant was mainly used to feed the AMF instead of for plant growth. It is also possible that part of the energy produced was directed to the plant-microorganism mutualistic induction of photoprotective and antioxidant mechanisms that preserved the PSII machinery under the detrimental conditions of nutrient limitation.

It is already known that plants share their photosynthetically fixed carbon with their associated microbial community, while microorganisms confer the ability to use previously nonavailable nutrients to their host plants, creating an important mechanism with which to resist abiotic stress (Smith et al., 2015; Bernardo et al., 2017; Lata et al., 2018). Therefore, our AMF-plant interactions under low nutrient conditions required energy to synthetize and share these biochemical compounds to resist abiotic stress and show a strong photosynthetic response (high ETR_MAX_, high NPQ values, and high polyphenol content). In this sense, although our mutualistic interaction experiment with tomato plants and AMF in a low-nutrient medium was found to be energetically expensive, it conferred to the plants the capacity to survive under harsh conditions through the assimilation of normally nonavailable nutrients from the soil and increased plant resistance to stress. This is supported by what was observed in the control plants given that control plants without any PBs or NS died in the early stages. Clearly, AMF mutualistic interactions confer an important ecological advantage to tomato plants under low-nutrient conditions.

SEs are comprised of a multifaceted mixture of bioactive compounds, including polysaccharides, fatty acids, vitamins, phytohormones, and mineral nutrients and may induce various positive physiological responses that are reflected in improved root length, biomass production of the aerial portion of the plant, and plant weight (Craigie, 2011; Battacharyya et al., 2015). In this study, we also observed physiological and ecological benefits in plants treated with the algal extract. The addition of SE enabled the plants to survive in low nutrient soil (i.e. sand). The most characteristic benefit of SE in this work was related to the promotion of root length. These results agree with those of Hernandez-Herrera et al. (2014a), who attributed their similar findings to the minerals present in the SEs. Also, Hernández-Herrera et al. (2014a; 2014b; 2016) reported that SE and polysaccharide-enriched extracts from *P. gymnospora* conferred strong root growth-promoting activity in tomato plants and resulted in mung bean hypocotyl cuttings with longer roots when compared with those of the control plants and plants treated with Indole-3-butyric acid, a synthetic rooting hormone. These results suggest that oligosaccharides may behave as signaling molecules that trigger changes in the endogenous phytohormone metabolism of treated plants by a selective regulation of associated genes (De Pascale et al., 2017).

It has also been shown that different SE treatments promote biotic and abiotic responses in tomato plants, including the activation of defense-related enzymes, such as SOD, POD, PPO, and PAL (Kumar and Pandey, 2013; Hernandez-Herrera et al., 2014b; Drobek et al., 2019) and phenolic compounds (El Modafar et al., 2012) as well as the accumulation of proline and soluble sugars (Goñi et al., 2018). According to Mzibra et al. (2020), the tomato plant cell wall may recognize the hydrolysis of SE polysaccharides that can behave as signaling molecules and induce phytochemical biosynthesis, including that of polyphenols. In this study, surprisingly, polyphenol content was high in the SE-treated plants when no nutrients were added, but low when nutrients were supplied *via* irrigation, and no accelerated flowering was detected with regard to what was observed with AMF addition. Therefore, results showing a 2-fold increase in polyphenol content in the SE-treated plants vs NS-treated plants in this study could be attributed to tomato plant growth under stressful conditions. In addition, SE + NS-treated plants showed significantly higher NPQ than that of the untreated plants with NS. These results agree with those of Santaniello et al. (2017) who reported similar NPQ results in SE-treated plants under dehydration conditions, supporting the idea that SE confers photoprotection under stressful environmental conditions.

The information available with regard to the influence of SEs on fungal growth, root colonization, and infection rates in tomato plants indicates that the potential mechanisms for stimulating AMF hyphal growth acted *via* various low-weight isolated compounds, such as 5-deoxy-5-methylamino-adenosine as well as alginic acid, mannitol, and some polysaccharides from various seaweed species (Kuwada et al., 1999; Ishii et al., 2000; Kuwada et al., 2005; Kuwada et al., 2006a; Kuwada et al., 2006b; Khan et al., 2009; Paszt et al., 2015), which led to increased phosphorus acquisition. In this study, the application of *Padina gymnospora* SE enhanced the growth of the lateral roots of un-mycorrhized plants and improved root colonization by AMF; however, no improvement was observed in phosphorus acquisition or accumulation. Thus, the enhanced fungal growth and root colonization may have been due to the alginic acid, alginate oligosaccharides, or mannitol contained in the SE, which has been observed in the brown seaweed *P. gymnospora* (Chennubhotla et al., 1977). Another possible explanation for the improvement in lateral root growth by the SE may have been due to a change in the hormonal balance as a consequence of the auxins contained in the SEs, which are root hair growth regulators that promote elongation through the up-regulation of associated root epidermis genes, and thus mycorrhization was consequently improved as a result of greater root development (Zhang et al., 2019). In the same way, the polyphenol and carbohydrate content in the *P. gymnospora* SE at 0.8% (**Supplementary Table S1**) may have been used by the AMF, which may have been reflected in the observed improvements in fungal growth and root colonization.

In particular, the combined effect of both PBs resulted in high values of the physiological parameters evaluated, although PB efficacy was only equal to the sum of the independent effects. Therefore, a clear additive interaction on the physiological parameters was observed when AMF and SE were applied simultaneously to the tomato plants. The plants grown under optimal conditions (irrigated with the NS) that were treated with AMF and SE showed high growth, high fresh biomass, and low ETR_MAX_. Clearly, the photoprotective and photorepair mechanisms as well as the costly alternative metabolic routes for nutrient assimilation were not necessary under less-stressful conditions when compared to those of the AMF-plants grown in sand and water. Therefore, under less-stressful environmental conditions, most of the fixed carbon could be redirected to maximize growth and development, which suggests an optimization of resources that is beneficial to both organisms of the mutual interaction (the plant and the mycorrhizal fungi). According to Rouphael et al. (2017), the application of combined PBs resulted in beneficial effects that could be associated with increased chlorophyll biosynthesis, a greater ability to maintain the photochemical activity of PSII, and a favorable nutritional status in leaf tissues compared with those of plants grown in the absence of combined PB addition.

In this experiment, the values of *F_V_/F_M_* found in all treatments can be considered slightly suboptimal. However, they may be associated with a natural response of self-reduction and dynamic photoinhibition due to the natural variation of environmental variables instead of to the chronic photoinhibition associated with PSII damage (Demmig-Adams et al., 2017). However, no detailed photoinhibition experiment was conducted. Further investigation is needed to assess the high photoprotection offered by AMF alone in low nutrient soils. The activation of the Xanthophyll cycle and acceleration of D1 PSII-core protein expression as well as the ability to detoxify ROS may constitute important mechanisms that help to improve physiological performance and ecological fitness, which require further study.

In this work, we analyzed some of the biochemical and ecological characteristics of the plants in each PB treatment. We identified that each PB (AMF or SE) affected the plants in a different manner. Based on both morphological and transcriptomic data, mycorrhization has been found to accelerate flowering and fruit development in tomato plants (Salvioli et al., 2012). Perhaps the most important benefit of the microbial AMF PB was related to early floriation, which is a promising finding that reflects the economic potential of the use of this PB to increase production. Also, AMF has been found to significantly increase tomato dry biomass and citric acid concentrations (Bona et al., 2017). In addition, AMF was found to increase the polyphenol concentration 6-fold. Both abiotic stress and fungal infections have been found to induce phenol and secondary metabolite accumulation in plant tissues (Zhi-Lin et al., 2007).

In contrast, SE increased protein accumulation, which agrees with what was found by Pise and Sabale (2010), who reported that the increases in protein content of SE-treated plants may have been the result of seedlings being able to absorb most required elements. Also, enhanced N uptake and translocation have been frequently associated with the highest protein content observed in PB-treated plants (Calvo et al., 2014). However, the total N content in the tomato plants in this study did not support this observation.

The combination of both PBs, enriched by the NS, resulted in a synergistic interaction of the PBs and was reflected in a favorable and significant enhancement of the biochemical (protein and carbohydrate levels) and ecological (AMF colonization and flowering) characteristics of the tomato plants. The elevated protein content may have been the result of amino acids that were directly used for protein biosynthesis after being incorporated by the plant. Nonetheless, increased protein content has been found to be associated with increased carbohydrate concentrations in plant leaves (Abbas, 2013). High sugar content in the leaves generally accelerates the nitrate assimilation pathway. Carbohydrates are required for the incorporation of ammonia in amino acids and accelerate the biosynthesis of proteins (Bulgari et al., 2015). Although chemical composition was measured in vegetative tissue in this experiment, it is highly probable that stable responses could be present in tomato fruits, in which PB application has been shown to shorten the ripening time (Yao et al., 2020). The results of this study are also consistent with those obtained by Faris (2013); Bettoni et al. (2014), and Rouphael and Colla (2018) who demonstrated that different PB combinations provided reproducible plant growth and production benefits, either in the form of additive or synergistic interactions.

When the ecological parameters were analyzed, such as mutual association success (AMF colonization) and flowering, a greater number of flowers were present on AMF-treated tomato plants compared to that of the SE-treated tomato plants. Flowering is driven by the requirements for carbon and nutrients and phytohormones, which may be affected by AMF (Torelli et al., 2000; Boldt et al., 2011). The AMF accelerated flowering and fruit production but also increased the amino acid concentration in the fruits, especially that of glutamine and asparagine (Salvioli et al., 2012). Poulton et al. (2002) demonstrated that AMF colonization can enhance host plant fitness by positively affecting its reproductive functions. In addition, SEs may improve flower yield and fruit production by behaving as chelators (Battacharyya et al., 2015; Yuvaraj and Gayathri, 2017; Pohl et al., 2019). Also, hormonal responses are mainly attributed to the cytokinins present in the SE or to their production, transport, and mobilization into developing tissues, either vegetative or floral (Ganesan et al., 2015). Similarly, Bona et al. (2017) reported that the farmed tomato plants treated with either AMF or PBs or their combination showed improved flower number and tomato fruit size and weight as well as enhanced industrial fruit (i.e., dry biomass, pH, and nitrate and citrate concentrations) and nutritional (i.e., sugar, ascorbate, and lycopene content) characteristics.

Additionally, applying PBs to plants has been shown to produce healthy plants with a significantly higher number of fruits and weights compared to those of plants grown in the absence of PBs (Hamed et al., 2018; Rouphael and Colla, 2018). In this study, we have shown that the addition of SE to AMF-treated plants accelerated flowering and resulted in a larger flower number compared to that of plants only supplied with AMF. Interestingly, the combined effect of both PBs resulted in high values of the physiological parameters evaluated, although the efficacy of the PBs was only equal to the sum of their independent effects. Therefore, a clear additive interaction on physiological parameters was observed when AMF and SE were applied simultaneously to the tomato plants. However, superior crop performance was related to enhanced nutrient availability, which was driven by the combined application of SE and AMF that resulted in a synergistic interaction. Understanding the effects of the different PBs, either alone or in combination, on the tomato plants in our study implies that we could select the appropriate PB to obtain preferred product characteristics, which constitutes an important biotechnological application.

Currently, most biostimulants are complex chemical mixtures that are either derived from or extracted from biological processes and materials. In fact, AMF and SE mixture complexity has been thought to be a critical determinant of how well the biostimulant performs. Moreover, PB properties may be the result of the collective sum of all of their components and may not be fully understood by evaluating either component characteristics independently or particular component combinations. These properties are known as “emergent properties” and cannot be fully understood from a functional decomposition analysis (Yakhin et al., 2017). These novel emergent properties arise when an elevated degree of structural complexity is attained with lower-complexity components.

Based on an analysis of all data *via* the PCoA, we have shown how scale-specific observations may not be reflective of responses at the whole-plant level. We have highlighted the limitations of classical individual analysis based on the empirical evidence generated in this study. Nevertheless, modularity (AMF or SE) or approaches that consider the whole as the sum of its parts (AMF + SE) are unable to produce a complete understanding of physiological, biochemical, and ecological implications of SE and AMF PBs in tomato plants. Indeed, the integration of modules allows for the emergent properties of biological systems to become known resulting in the creation of unique individual entities. Therefore, we can adopt the term “emergent properties” in this work to describe the appearance of novel properties due to a particular degree of structural complexity forming from multiple components. The combined application of AMF and SE resulted in responses that could not be identified through functional decomposition. By identifying additive and synergistic results as well as additional effects from the combined use of both PBs, we suggest that beneficial, novel, and emergent properties arise from the constituent complex that forms following the application of both PBs and not as the result of the independent effects of known essential plant nutrients, growth regulators, or protective compounds.

## Conclusion

When AMF and SE PBs were used individually, each was found to positively stimulate plant growth and yield in a different but complementary manner, since AMF promoted foliar growth while SE promoted root enhancement. However, the benefits of the combined application of both AMF and SE on crop growth and yield should be emphasized. SE was found to boost the AMF population; therefore, the benefits of the combined application of the PBs were additive with regard to the physiological variables but synergistic with regard to the ecological and productivity related parameters. The main advantages of the AMF and SE PBs were reflected in positive impacts on plant quality and performance. The findings of this study have identified patterns in complex data and elucidated the inherent activity and synergy of potential microbial and nonmicrobial PBs that may be used in commercial agriculture.

According to the data provided by FAOSTAT, 182,301,395 tons of tomatoes were produced globally in 2017, the cultivation of which is highly dependent on mineral nutrition (FAOSTAT, 2019). Mexico produces 4,243,058 tons of tomatoes annually, and utilizes 92,993 ha of land for tomato plantations. In fact, Mexico is the top tomato exporting country in the world, accounting for approximately 24.5% of the total tomato exports worldwide. Unfortunately, data of tomatoes produced with the use of biostimulants does not exist. In Mexico, the *Norma Oficial Mexicana* (NOM-077-FITO-2000) enables the Secretary of Agriculture and Rural Development to regulate phytosanitary and plant nutritional aspects of agricultural production, establishing the specifications, criteria, and procedures to regulate studies of the biological effectiveness of plant nutrition inputs. “The plant nutrition inputs provide essential elements to stimulate the growth and development of plants, correct or prevent any nutritional deficiency, or temporarily improve the properties of the soil, in order to increase the yield and quality of agricultural products” (NOM-077-FITO-2000, 2000). These inputs are highly diverse (e.g., organic and organic-mineral fertilizers, organic soil improvers, inoculants, soil humectants, and types 1, 2, and 3 growth regulators) and many have been developed with the intention to be registered and commercialized in Mexico, making it necessary to demonstrate their effectiveness in the field. Specifically, biological effectiveness is measured when applying a plant nutritional input, which may be said to be a biostimulant if it improves nutritional uptake efficiency, tolerance to abiotic stress, and/or crop quality. In Mexico, the production and use of biostimulants is still limited, and PBs have rarely been incorporated into the established cultivation practices, which is in part due to a lack of understanding on behalf of farmers regarding biostimulant functions and application. This gap in understanding has resulted in a hesitancy to use biostimulants based on a fear of additional cultivation costs and a reduction in the quality and quantity of plants and overall crop profitability. Therefore, it is important to introduce a truthful understanding of PBs to replace the use of organic fertilizers and agrochemicals. Biostimulants have negligible nutrient concentrations and act on the metabolism of a plant, unlike fertilizers. PBs may facilitate nutrient acquisition and translocation by enhancing metabolic processes that take place in the soil and in plants. For example, AMF development or the addition of highly diluted SE solutions induce the mineral exchange of P and N found in the soil, making these nutrients available to plants (De Pascale et al., 2017; Lucini et al., 2019; Shukla et al., 2019).

An elucidation of the functions of AMF and SE as first-generation PBs, when they are applied either alone or in combination, would allow for the development of a second generation of biostimulants. Specifically, understanding improved nutrient use efﬁciency, plant quality, and tolerance to abiotic stress will allow for the development of second-generation PBs with specific synergistic and complementary biostimulant actions, the mechanisms of which could be functionally designed. In agriculture, the application of both microbial and nonmicrobial PBs could substantially promote sustainability efforts. Consequently, greater collaboration between farmers, industrial sectors, researchers, and governmental entities is required to improve production systems and PB quality to create and implement improved and environmentally friendly agricultural practices.

## Data Availability Statement

The original contributions presented in the study are publicly available. This data can be found in the TRY Plant Trail Database: https://www.try-db.org/TryWeb/Data.php#56.

## Author Contributions

Conceptualization: RH-H. Data curation: MG-G and RH-H. Formal analysis: HO. Funding acquisition: RH-H. Investigation: MG-G. Methodology: HO-A, CS-H, and FS-R. Project administration: RH-H. Resources: FS-R, CS-H, and KC-C. Software: AB-E, HO-A, and JC-N. Supervision: RH-H and HO-A. Validation: FS-R. Visualization: RH-H and HO-A. Writing—original draft: RH-H and HO-A. Writing—review and editing: RH-H and HO-A.

## Funding

This work was supported by the project number UDG-PTC-1329 of PRODEP to RH-H; UDG-PTC-1460 of PRODEP to HO-A; Universidad de Guadalajara by PROSNI-2017 and P3E to HO-A and PROSNI-2019 and P3E to RH-H.

## Conflict of Interest

The authors declare that the research was conducted in the absence of any commercial or financial relationships that could be construed as a potential conflict of interest.

## Footnotes

1 https://www2.dijon.inrae.fr/mychintec/Mycocalc-prg/download.html
