# Supplementary material for: Physiological, Ecological, and Biochemical Implications in Tomato Plants of Two Plant Biostimulants: Arbuscular Mycorrhizal Fungi and Seaweed Extract
Source: Front Plant Sci. 2020 Jul 17;11:999. doi: 10.3389/fpls.2020.00999 (PMC7379914; doi:10.3389/fpls.2020.00999)
Supplement: Supplementary file 1 [file DataSheet_1.docx]

Supplementary Material

# Supplementary Tables

**Supplementary Table 1.** Chemical composition of seaweed extract (SE) from *Padina gymnospora*

| **Variables** | **Value** |
| --- | --- |
| pH | 5.92 |
| Electric conductivity (dS m^−1^) | 0.25 |
| Total solids (%) | 0.20 |
| Organic matter (%) | 0.02 |
| Density (g mL^−1^) | 1.00 |
| Ash (%) | 0.17 |
| Polyphenols (mg L^−1^) | 10.57 |
| Carbohydrates (mg L^−1^) | 41.76 |
| **Macronutrients** | **(%)** |
| Organic carbon | 0.014 |
| Total nitrogen | 0.019 |
| C/N Ratio (10:1) | 0.73 |
| Total phosphorous | < 0.0001 |
| Phosphorous (P_2_O_5_) | < 0.0001 |
| Total potassium | 0.07 |
| Potassium (K_2_O) | 0.08 |
| Total calcium | 0.0011 |
| Total sodium | 0.538 |
| Chloride | 0.0391 |
| Sulfates (S-SO_4_) | 0.0327 |
| Bicarbonate (HCO_3_) | 0.0049 |
| **Micronutrients** | **(%)** |
| Iron | 0.0002 |
| Zinc | 0.0001 |
| Copper | 0.00001 |
| Boron | 0.00016 |

**Table S2.** Rorison’s nutrient solution composition (Hewitt, 1966)

| Element | Form | Concentration  (mg⋅L^−1^) |
| --- | --- | --- |
| Mg | MgSO_4_·7H_2_O | 248.0 |
| Ca | Ca (NO_3_)_2_·4H_2_O | 476.0 |
| K | K_2_HPO_4_·3H_2_O | 50.0 |
| P | K_2_HPO_4_ | 50.0 |
| Fe | EDTA– FeSO_4_·7H_2_O | 250.0 |
| Mn | MnSO_4_·4H_2_O | 2.24 |
| Mo | (Na_4_)_6_ Mo_7_ O_24_ • 4H_2_O | 3.0 |
| B | H_3_BO_3_ | 2.88 |
| Zn | ZnSO_4_·7H_2_O | 0.44 |
| Cu | CuSO_4_·5H_2_O | 0.40 |
| N | (Ca (NO_3_)_2_·4H_2_O | 476.0 |

**Supplementary Table S3**. Primers used for qRT-PCR analyses

| **Accession number** | **Gene** | **Protein** | **Primer sequence (5’- 3’)** |
| --- | --- | --- | --- |
| AY885651 | *LePT4* | Phosphate transporter | GAAGGGGAGCCATTTAATGTGG |
|  |  |  | ATCGCGGCTTGTTTAGCATTTC |
| DQ282611 | *RiEF1-α* | Elongation factor 1-alpha | GCTATTTTGATCATTGCCGCC |
|  |  |  | TCATTAAAACGTTCTTCCGACC |
| SGN-U316474 | *SAND* | SAND family protein | TTGCTTGGAGGAACAGACG |
|  |  |  | GCAAACAGAACCCCTGAATC |

**Supplementary Table S4.** Loading values of the morphometric and physiological variables identified by the principal coordinate analysis (PCoA) for tomato plants inoculated with arbuscular mycorrhizal fungi (AMF) and treated with seaweed extract (SE).

| Variable | PCO1 | PCO2 | PCO3 |
| --- | --- | --- | --- |
| Shoot length | -0.572 | 0.693 | -0.415 |
| Root length | -0.693 | -0.559 | 0.306 |
| Leaf area | -0.940 | 0.339 | -0.048 |
| Root area | -0.955 | -0.089 | 0.277 |
| Limb | -0.561 | 0.014 | -0.370 |
| Leaf | -0.528 | 0.430 | -0.163 |
| Fresh weight | -0.978 | 0.195 | 0.009 |
| ETR | 0.875 | 0.364 | 0.318 |

**Supplementary Table S5.** Loading values of the ecological variables identiﬁed by principal coordinate analysis (PCoA) for tomato plants treated with arbuscular mycorrhizal fungi (AMF) and treated with seaweed extract (SE).

| Variable | PCO1 | PCO2 | PCO3 |
| --- | --- | --- | --- |
| Phosphorous | -0.913 | -0.181 | 0.040 |
| Number of flowers | -0.811 | -0.584 | -0.036 |
| Root mycorrhizal colonization intensity (M%) | -0.999 | 0.041 | 0.005 |
| Polyphenols | -0.601 | 0.789 | -0.131 |
| Non-photochemical quenching (NPQ) | -0.521 | 0.723 | 0.344 |
